# Downregulation of PTCD1 in Bladder Urothelial Carcinoma Predicts Poor Prognosis and Levels of Immune Infiltration

**DOI:** 10.1155/2022/1146186

**Published:** 2022-06-28

**Authors:** Zhongbao Zhou, Yongjian Zhou, Xin Zhou, Yongjin Huang, Yuanshan Cui, Yong Zhang

**Affiliations:** ^1^Department of Urology, Beijing Tiantan Hospital, Capital Medical University, Beijing 100070, China; ^2^Department of Urology, The Affiliated Yantai Yuhuangding Hospital of Qingdao University, Yantai, Shandong, China

## Abstract

Pentatricopeptide repeat domain 1 (PTCD1) was reported to regulate mitochondrial metabolism and oxidative phosphorylation. However, the effect and mechanism of PTCD1 in the development of bladder urothelial carcinoma (BLCA) remain unclear. The databases from The Cancer Genome Atlas (TCGA) and Human Protein Atlas (HPA) were used to analyze the expression changes, clinical features, and prognostic values of PTCD1. A nomogram was built to predict the prognostic outcomes of BLCA cases. The potential genes interacting with PTCD1 were explored by Weighted Gene Coexpression Network Analysis (WGCNA). The estimation of associations between PTCD1 and tumor mutations, tumor immunities, and m6A methylations was performed. The study found that the gradual decrease of PTCD1 expression was observed with the increase of stage and grade. Low PTCD1 expression was greatly correlated with higher pathological stage, N stage, and poor prognosis in TCGA cohorts; interestingly, low-grade BLCA cases all exhibited high expression of PTCD1. HPA database analysis implied that the expression of PTCD1 protein in BLCA was lower than that in normal bladder tissue, and the protein expression of PTCD1 in high-grade BLCA was lower than that in low-grade BLCA. Multivariate Cox regression analysis indicated that PTCD1 may serve as an independent factor influencing prognosis of BLCA. Mechanistically, PTCD1 played a regulatory role in BLCA progression through multiple tumor-related pathways containing PI3K-Akt signaling, ECM-receptor interaction, oxidative phosphorylation, and extracellular matrix organization. WGCNA reported that PTCD1 had a strong positive correlation with POLR2J, ZNHT1, ATP5MF, PDAP1, BUD31, and COPS6. Besides, the mRNA expression of PTCD1 was negatively associated with immune cells' infiltrations, immune functions, and checkpoints, especially with some m6A methylation regulators in BLCA. In sum, downregulation of PTCD1 expression may be involved in the development of BLCA and remarkably correlated with poor prognosis. Meantime, it showed an influence in immune cell infiltration and may serve as an agreeable prognostic indicator in BLCA.

## 1. Introduction

Bladder urothelial carcinoma (BLCA) is considered as one of the most invasive cancers in the world [1]. It is reported that the five-year overall survival (OS) in BLCA is approximately 80% [2]. Furthermore, it is estimated that more than 80 thousand new cases and 17 thousand deaths annually occur in the United States alone [3]. Bladder tumors are assorted into high and low grade according to histomorphological features following the description from the World Health Organization [4]. About eighty precent of BLCA cases express non-muscle-invasive bladder cancer (NMIBC) in the diagnosis, while the rest cases show muscle-invasive bladder cancer (MIBC) or even distant metastasis [5]. NMIBC generally does not pose threats to the survival of patients and has better prognosis for effective treatment options [6]. However, T1 tumors in NMIBC act as an invasive subtype with forty percent relapse and fifteen percent progression to MIBC within five years [7]. MIBC has clinical aggression with a rapid development of metastases to bone, brain, liver, lungs, and lymph nodes, which usually poses a fatal threat to patients [8]. Despite significant progress in revealing the molecular mechanisms and surgery techniques of BLCA in recent years, there is no significant decrease for the mortality rate [9–11]. Therefore, exploring molecular biomarker is helpful to the early disease diagnosis, progression, and prediction of prognostic and individualized therapy for specific patient [12].

Pentatricopeptide repeat (PPR) proteins are a large class of RNA-binding proteins characterized by a typical thirty-five residue repeat motif [13]. Only seven PPR proteins are found in human beings, all of which are located in mitochondria and involved in various cell procedures, notably in RNA supersession [14, 15]. The discovery of essential role of PPR proteins in mitochondria gene expression and energy metabolism underscores its biological importance [15]. For example, mitochondrial RNA polymerase regulates the transcription of mitochondrial mRNA (mt-mRNA) [16]. PPR domain 1 (PTCD1), PPR domain 2 (PTCD2), and only protein RNase P catalytic subunit are related to the process of mt-mRNA [17, 18]. Leucine-rich pentatricopeptide repeat motif-containing protein (LRPPRC) is involved in the evolution and stability regulations of mt-mRNA, and PPR domain 3 (PTCD3) is related in protein composite in mitochondria [19, 20]. In recent years, an increasing number of PPRs have been identified with important regulation effects of BLCA carcinogenesis. For instance, LRPPRC promotes BLCA tumorigenesis by regulating redox homeostasis through the circANKHD1/FOXM1 [15]. Black et al. reported that FOXM1 translocated into mitochondria and inhibited mitochondria respiration and oxidative phosphorylation by enhancing PTCD1 and may be a promising molecular target [21]. As above shown, PTCD1 is very likely involved in formation and development of cancer. However, the effect of PTCD1 in BLCA is still not well clarified.

In this research, RNA-seq data obtained from The Cancer Genome Atlas (TCGA) database was employed to comprehensively assess the diversities in PTCD1 expression and their relationships with patient outcomes. The Human Protein Atlas (HPA) database was used to explore the protein expression of PTCD1 in normal bladder and tumor tissues. Moreover, Gene Set Enrichment Analysis (GSEA) was conducted on the high- and low-PTCD1 expression groups, respectively, to show the latent mechanisms. Next, we explored the potential genes interacting with PTCD1 by Weighted Gene Coexpression Network Analysis (WGCNA). Finally, the association between the expression of PTCD1 and extent of immune cell infiltration was investigated to examine the probable mechanism of PTCD1-induced tumorigenesis and development.

## 2. Materials and Methods

### 2.1. Gathering of PTCD1 Expression Data from TCGA and HPA Databases

We followed the methods of Dr. Sun et al. [22]. The expression of PTCD1 combined clinical data (containing 414 BLCA cases) were gathered from TCGA database (https://portal.gdc.cancer.gov/). The clinical data mainly included age, sex, grade, pathological stage, TNM stage, survival condition, and survival time. The exclusion criteria were as follows: (a) The pathological diagnosis did not meet BLCA; (b) The PTCD1 expression profile and clinical data were incomplete. We used the HPA database (https://www.proteinatlas.org/) to explore the protein level of PTCD1 in tissues. Normalization was performed by log 2 (FPKM + 1) to transform expression data for data analysis.

### 2.2. Survival Analysis

Survival data were analyzed using R software and R package “survival” and “survminer.” The threshold was identified by the best cut-off expression of PTCD1 in tumor samples and the queues were classified into low- and high-expression groups. The correlations between PTCD1 expression and patient outcomes, including OS, grade, and stage, were examined. The ENCORI database (https://starbase.sysu.edu.cn/) was applied to verify the prognosis of PTCD1 in BLCA.

### 2.3. Diagnostic Value and Construction of Prognostic Nomogram

The univariate and multivariate Cox regression analyses were used to assess whether PTCD1 expression served as an independent prognostic factor in BLCA using “glmnet” package in R software. Based on the results of multivariate Cox regression analysis, we performed a prognostic nomogram to quantify the prediction of prognosis for BLCA patients. The concordance index (C-index) and calibration curve were employed to assess the reliability and accurateness of nomogram. In addition, we evaluated the effects of PTCD1 expression on the clinical prognosis in BLCA patients.

### 2.4. Differentially Expressed Genes and Functional Analysis

Background correction and normalization were performed on public microarray data, and R package “limma” was applied to identify differential expression genes (DEGs) between low- and high-PTCD1 expression groups. The *P* values were corrected for multiple test correction by false discovery rate (FDR). DEGs were included in the Gene Ontology (GO) term enrichment analysis and Kyoto Encyclopedia of Genes and Genomes (KEGG) pathway analysis using the R package “clusterProfiler,” which were recruited when |log2FC| value is more than 1 and FDR less than 0.001. FDR *q*-value less than 0.05 and *P* adjustment less than 0.05 were greatly enriched for gene sets.

### 2.5. WGCNA and Module Identification

WGCNA was employed to determine the gene module closely related with PTCD1 expression. To identify gene clusters that were likely to be highly coexpressed, a coexpression network was constructed using profiles of DEGs dataset with R package “WGCNA.” First, cluster analysis was performed on tumor samples using the “hclust” function to check and remove outliers. Second, Pearson's correlation between every pair of extracted gene was analyzed to generate an adjacency matrix. Third, a soft-threshold parameter performance value (*β*) was built, which can accentuate the strong correlation of genes while penalizing the low correlation to guarantee the construction of a scale-free network. Furthermore, based on TOM-based dissimilarity (1-TOM) with a dendrogram of more than 30 genes, hierarchical clustering analysis was conducted to examine modules with genes of similar expression profiles. A cutoff (less than 0.25) was then chosen to incorporate the similar modules to ensure the outcomes trustier. “DynamicTreeCut” algorithm was employed to construct networks and detect consensus modules. The relationships between modules and BLCA were computed by the module-trait associations with WGCNA. The module eigengene (ME, representing the gene expression profile of a module) was considered as the first major component of a given module. Finally, clinically significant modules were identified by computing the association between clinical features and MEs. By selecting modules where PTCD1 was located, the network construction and core gene screening were carried out.

### 2.6. Tumor Mutation Profile Analysis

We used the somatic mutation data obtained from the TCGA database and processed in R software using “maftools” package to identify the somatic variants and visualize somatic landscape. Mutant signatures from BLCA tissues were described with “Somatic Signatures” package in R software.

### 2.7. Relationships between PTCD1 Expressions and Immune Features

Immune cell infiltration data were gathered from TIMER website (https://timer.cistrome.org/). “GSVA,” “limma,” and “GSEABase” packages were employed to analyze and visualize the data. The relationships between different PTCD1 expression groups and immune infiltrations were examined by Wilcoxon rank-sum test. Immune function analysis of BLCA was conducted using the single-sample GSEA function with the R package “GSVA.” Moreover, the association between PTCD1 expression and immune checkpoint biomarkers was shown by Wilcoxon rank-sum test.

### 2.8. Correlation between PTCD1 Expression and m6A RNA Methylation Regulators

The matrixes of expressions were taken from transcriptome profiles datasets, containing regulators on writers (VIRMA, METTL3, METTL14, WTAP, RBM15, RBM15B, METTL16, ZC3H13, and PCIF1), readers (TRMT112, ZCCHC4, NUDT21, CPSF6, CBLL1, SETD2, HNRNPC, HNRNPG, HNRNPA2B1, IGF2BP1, IGF2BP2, IGF2BP3, YTHDC1, YTHDF1, YTHDF2, YTHDF3, YTHDC2, SRSF3, SRSF10, XRN1, FMR1, NXF1, and PRRC2A), and erasers (FTO, ALKBH5, and ALKBH3). The analysis of m6A RNA methylation regulators was conducted between the two differential PTCD1 expression groups using “limma” package with the Wilcoxon rank-sum test. *P* less than 0.05 was used as the significant significance.

### 2.9. Statistics Analysis

All statistical analyses were performed using R software (version 4.0.2). Wilcoxon rank-sum test was used to evaluate the difference of PTCD1 expression among multiple stratified clinical indicators. Categorical variables were presented as proportions, and continuous variables were expressed as mean ± standard deviation (SD). The association of clinicopathological variables in BLCA patients between high- and low-PTCD1 cohorts was subjected to a chi-square test. The survival curves were generated via the log-rank test for Kaplan–Meier analysis. The univariate cox regression model was used to analyze the effects of individual variables on survival, and the multivariate cox regression model was used to confirm independent factors associated with survival. Wilcoxon rank-sum test was applied to analyze the relationships between PTCD1 expression and molecular functions. The difference was considered significant when *P* < 0.05.

## 3. Results

### 3.1. The Expression Characteristics of PTCD1 in BLCA

**Figure 1:**
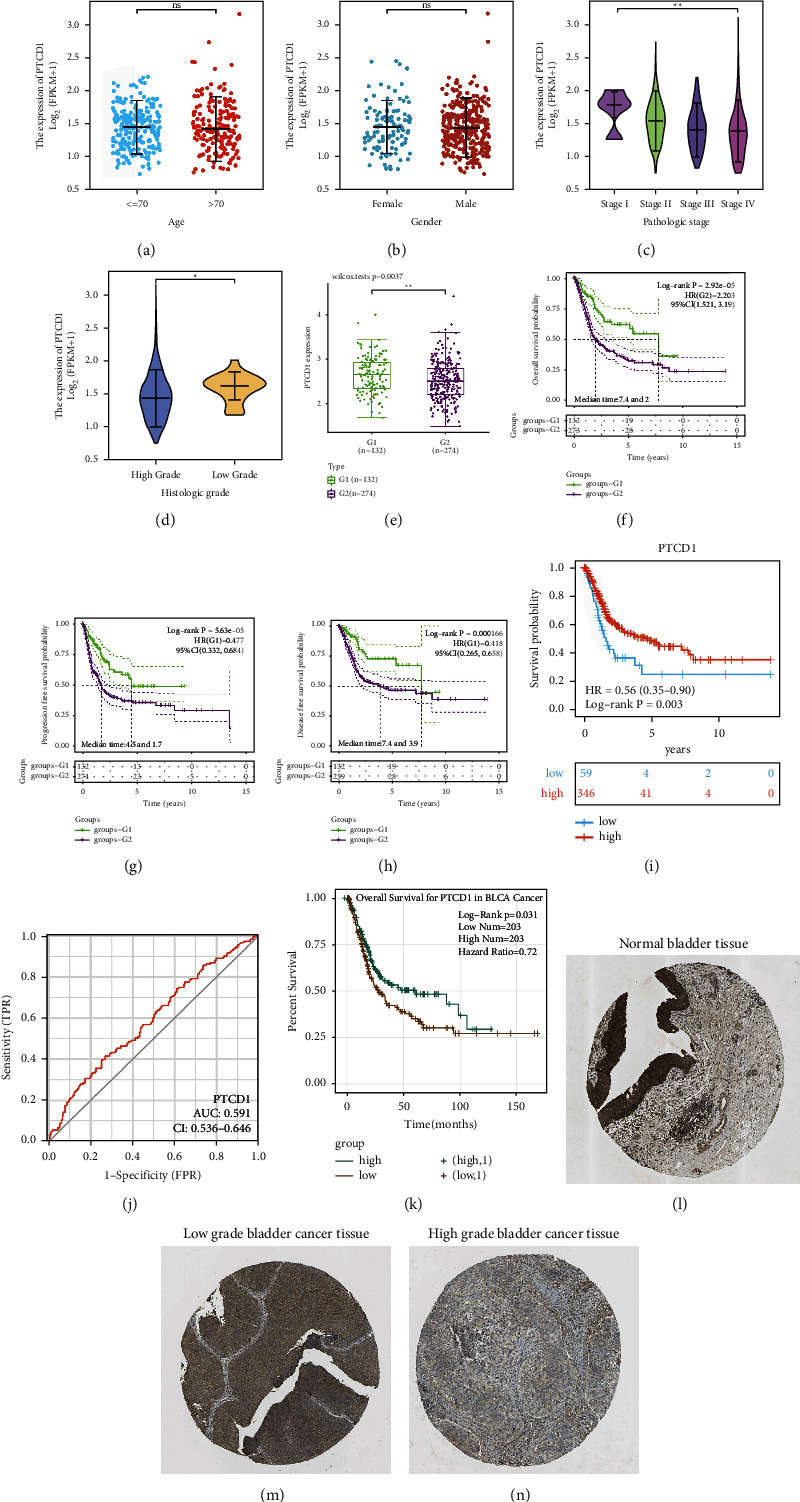
The PTCD1 expression level and survival analysis in BLCA. (a) The PTCD1 expressions of patients with BLCA according to different clinical characteristics including age (a), gender (b), stage (c), and grade (d). The PTCD1 expression (e), OS (f), PFS (g), and DFS (h) of stages I-II versus stages III-IV in the TCGA-BLCA dataset. The K-M curve between low-PTCD1 group and high-PTCD1 group in the TCGA database (i) and AUC curve related to OS (j). The survival curve based on the median expression of PTCD1 as the threshold in ENCORI database (k). Protein expression of PTCD1 in normal bladder tissue (l), low-grade (m) and high-grade (n) bladder cancer. ^*∗*^*P* < 0.05; ^*∗∗*^*P* < 0.01; and ^*∗∗∗*^*P* < 0.001.

The stratified analysis according to clinicopathological characteristics was conducted to evaluate the difference of PTCD1 expression, including age (less than 70 years old and more than 70 years old), sex (male and female), stage (stage I, stage II, stage III, and stage IV), and grade (high grade and low grade), which showed that the expression of PTCD1 gradually reduced with the growth of stage (*P*=0.003), and the expression of PTCD1 in low-grade bladder cancer was importantly higher than that in high-grade bladder cancer (*P*=0.012), but age (*P*=0.428) or gender (*P*=0.996) had no effect on the expression of PTCD1 (Figures 1(a)–1(d)).

### 3.2. Relationship between PTCD1 Expression and Prognosis

**Figure 2:**
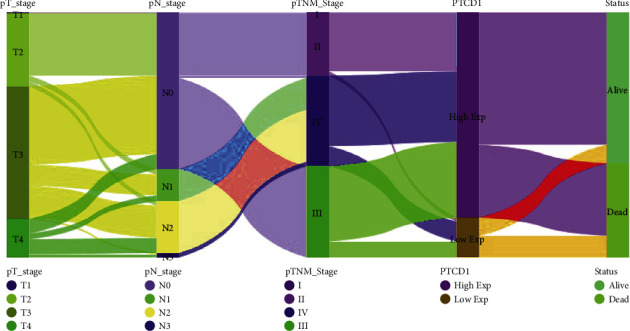
The Sankey diagram showed the connection between TNM stage, PTCD1 expression, and survival status, which showed that patients with high expression of PTCD1 tended to survive more.

We divided the patients in TCGA-BLCA data set into G1 (stages I-II) and G2 (stages III-IV) and found that the expression of PTCD1 in G2 group was importantly lower than that in G1 group (Figure 1(e)). Great differences were shown in OS (*P*=2.92*e* − 05), progression-free survival (PFS; *P*=5.63*e* − 05), and disease-free survival (DFS; *P*=0.000166) between the two groups (Figures 1(f)–1(h)). Next, TCGA-BLCA cohorts were classified into low- or high-PTCD1 groups based on the best cut-off expression of PTCD1 (FPKM = 1.128054) in tumor samples as the threshold. The features of BLCA patients were available in Table 1, and we found that the expression of PTCD1 was correlated with pathologic stage (*P*=0.001), N stage (*P*=0.031), and survival status (*P*=0.007) but was not associated with age (*P*=0.626), sex (*P*=0.851), grade (*P*=0.057), T stage (*P*=0.193), and M stage (*P*=0.648). Taken together, these results elucidated that the low expression of PTCD1 was greatly associated with advanced N stage and pathologic stage in BLCA; impressively, low-grade BLCA cases all exhibited high expression of PTCD1. The survival curve revealed that the survival ability of cases in the high-PTCD1 group was remarkably higher than that of the low-PTCD1 group (*P*=0.003) (Figure 1(i)). The area under curve (AUC) for OS reached 0.591, implying a good predictive value (Figure 1(j)). The ENCORI database also further confirmed the role of PTCD1 in the survival and prognosis of BLCA (*P*=0.031; Figure 1(k)). HPA database analysis implied that the expression of PTCD1 protein in BLCA was lower than that in normal bladder tissue, and the protein expression of PTCD1 in high-grade cases was lower than that in low-grade cases (Figures 1(l)–1(n)). Sankey diagram showed the interrelation between TNM stage, PTCD1 expression, and survival status, which showed that most patients with high expression of PTCD1 were in alive status (Figure 2).

### 3.3. Construction and Evaluation of Nomogram

**Figure 3:**
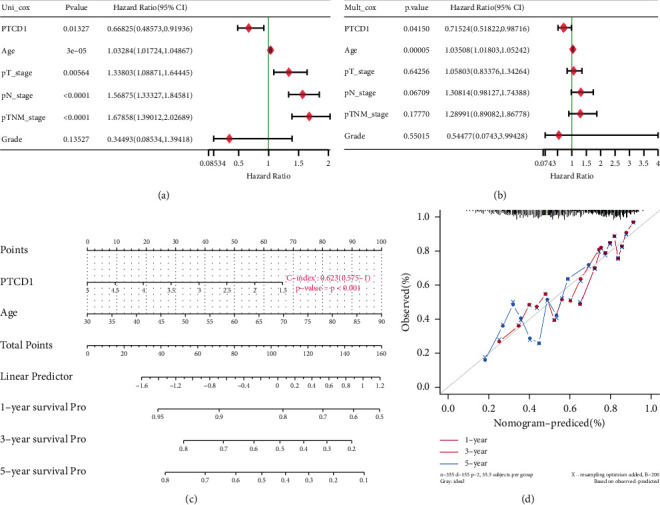
(a) Univariate Cox regression analysis; (b) multivariate Cox regression analysis; (c) nomogram for predicting probability of patients with 1-, 3-, and 5-year OS; and (d) actual and predicted survivals by the calibration curves.

The univariate and multivariate Cox regression analysis showed that PTCD1 expression [*P*=0.0411; hazard ratio (HR) = 0.715] and age (*P* < 0.001; HR = 1.035) were independent risk factors for survival prognosis of BLCA (Figures 3(a)-3(b)). The PTCD1 expression and patient's age were applied to construct a nomogram using the “rms” package in R software to predict 1-, 3-, and 5-year OS of BLCA patients (Figure 3(c)). The higher the PTCD1 expression of tissues, the better the prognosis of patients. The calibration curves were used to observe whether the actual prognostic value was consistent with the predicted value of nomogram, and it was found that the calibration curve of 1-, 3-, 5-year OS was almost consistent with the nomogram (Figure 3(d)).

### 3.4. Difference Analysis and Functional Analysis Based on Low- and High-PTCD1 Expression

**Figure 4:**
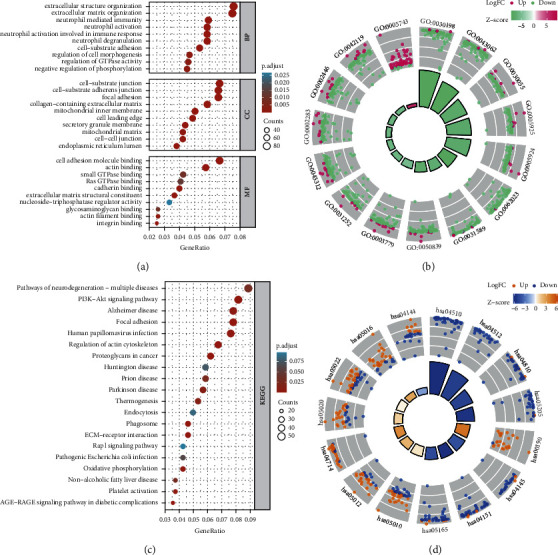
(a, b) Enriched biological process (BP), cellular component (CC), and molecular function (MF) of the DEGs. (c, d) Enriched Kyoto Encyclopedia of Genes and Genomes (KEGG) pathways of the DEGs.

Finally, 1,445 DEGs between low-PTCD1 group and high-PTCD1 group were determined, which were expressed in the Supplementary File 1. Next, we used the GSEA analysis to investigate the molecular mechanisms linked to the DEGs (Supplementary File 2). The highly abundant GO term in the biological process (BP) category was “extracellular matrix organization,” “extracellular structure organization,” “neutrophil mediated immunity,” and “cell-substrate adhesion.” Remarkably abundant GO terms associated with the cellular component (CC) category contained “cell-substrate junction,” “focal adhesion,” and “collagen-containing extracellular matrix.” In the molecular function (MF) category, DEGs were most abundant in the terms “actin binding,” “cadherin binding,” and “extracellular matrix structural constituent” (Figures 4(a) and 4(b)). Furthermore, KEGG pathway analysis indicated that these genes were especially correlated with PI3K-Akt signaling, focal adhesion, proteoglycans in cancer, ECM-receptor interaction, and oxidative phosphorylation (Figures 4(c) and 4(d)).

### 3.5. WGCNA and Module Identification

**Figure 5:**
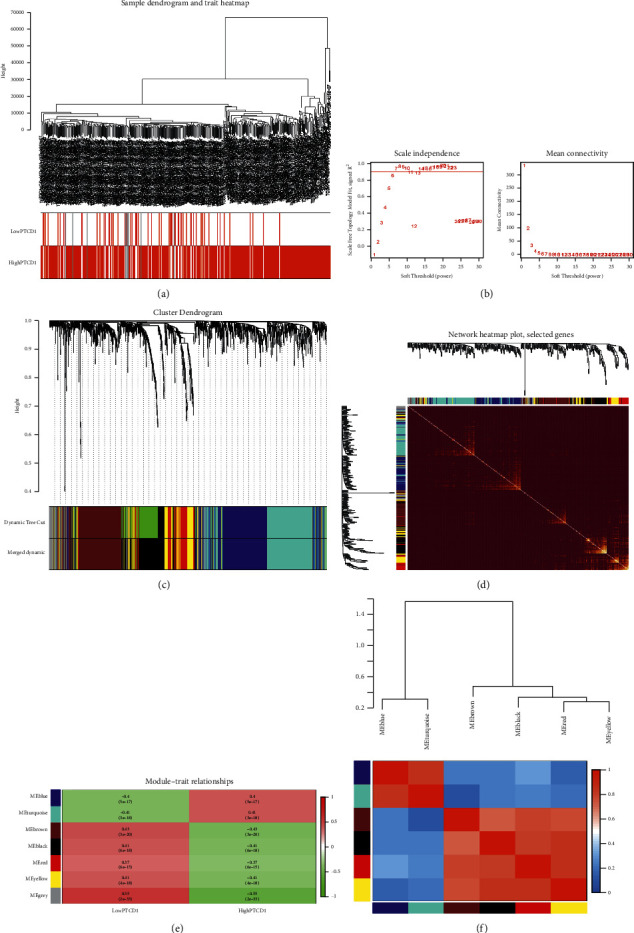
Identification of the module related with PTCD1 in DEGs dataset. (a) Clustering dendrograms of samples as well as traits; (b) the left panel shows the scale-free fitting indices for various soft-thresholding powers (*β*); (c) cluster dendrogram of coexpression network modules based on the 1-TOM matrix; (d) the gene interaction network of all modules; (e) heatmap of the correlation between module eigengenes and traits of BLCA; and (f) correlation heatmap between each module. Each module represents a cluster of corelated genes and was assigned a unique color.

To identify the module associated with PTCD1 expression, coexpression analysis was conducted to build the gene coexpression networks from DEGs dataset. Pearson's correlation and average linkage methods were used to cluster tumor samples of DEGs dataset (Figure 5(a)). No abnormal samples were detected or rejected. Optimal *β* = 6 (scale-free *R*_2_ = 0.9) was chosen to make sure to construct scale-free networks in DEGs dataset (Figure 5(b)). A cutoff of 0.25 and minimal module size of 30 and six modules in DEGs dataset (Figure 5(c)) were used to retain for consecutive analyses (gray modules indicate no assignment to any cluster). The gene interaction network of all modules was shown in Figure 5(d). We then examined the relationship between modules and traits by the heatmap (Figure 5(e)). Among them, the turquoise module including 290 DEGs was where PTCD1 was located, which were positively associated with the blue module (Figure 5(f)).

### 3.6. Coexpression Network Construction and Linear Correlation Analysis

**Figure 6:**
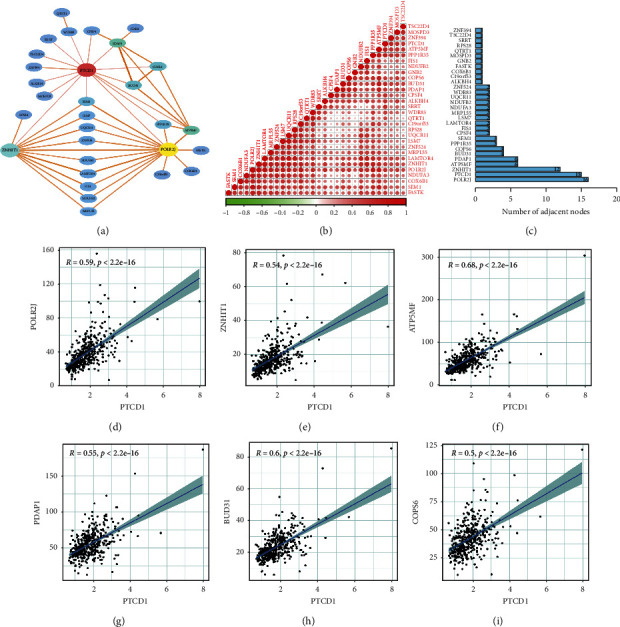
(a) Coexpression network of PTCD1 in the turquoise module. (b) Dot heatmap of gene correlation in coexpression network. (c) The bar chart showed the number of connecting nodes of target mRNAs in network. The association of PTCD1 with top six core genes including POLR2J (d), ZNHT1 (e), ATP5MF (f), PDAP1(g), BUD31 (h), and COPS6 (i).

We selected DEGs directly related to PTCD1 in the turquoise module to construct the expression network by Cytoscape v3.6.0 software (Figure 6(a)). The dot heatmap showed the strength of interaction of each gene (Figure 6(b)). We determined the number of adjacent nodes for each gene and visualized the associated role of the top six core genes with PTCD1 (Figure 6(c)), which identified that PTCD1 had a higher coexpression relationship with POLR2J (*R* = 0.59), ZNHT1 (*R* = 0.54), ATP5MF (*R* = 0.68), PDAP1(*R* = 0.55), BUD31 (*R* = 0.6), and COPS6 (*R* = 0.5), suggesting their potential interaction.

### 3.7. The Correlation between PTCD1 Expression and Somatic Mutation Load

**Figure 7:**
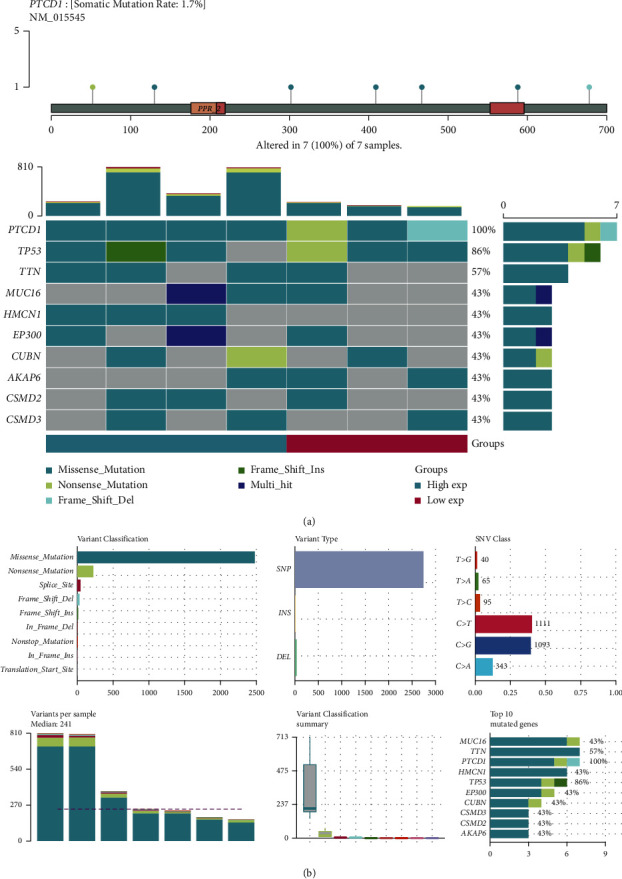
Mutation feature of PTCD1 in BLCA. The alteration frequency with mutation type and mutation site of PTCD1 (a). Distribution of frequently mutated genes in Patients with PTCD1 mutation. The upper bar plot shows the tumor mutation burden (TMB) for each patient, whereas the left bar plot indicates the gene mutation frequency in different groups (a). The top 10 BLCA-correlated mutation genes in patients with PTCD1 mutation and the mutation frequency, variant classification, variant type, and SNV class of the mutated genes in high- and low-PTCD1 subgroups (b).

By analyzing somatic mutation load data, we found that only 7 BLCA patients had PTCD1 mutation. The further analysis of relationship between somatic mutation load and PTCD1 expression in 7 samples was performed, indicating dominant type of variation classification was missense mutation (Figure 7(a)). A detailed comparison showed that single-nucleotide variant (SNV) was more common than insertions or deletions (Figure 7(b)). Furthermore, main SNV class showed the results of C > T and C > G (Figure 7(b)). Moreover, we ranked the top ten mutated genes in seven cases with calculated percentages (Figure 7(b)).

### 3.8. The Relationship between PTCD1 Expression and Immune Cell Infiltration

**Figure 8:**
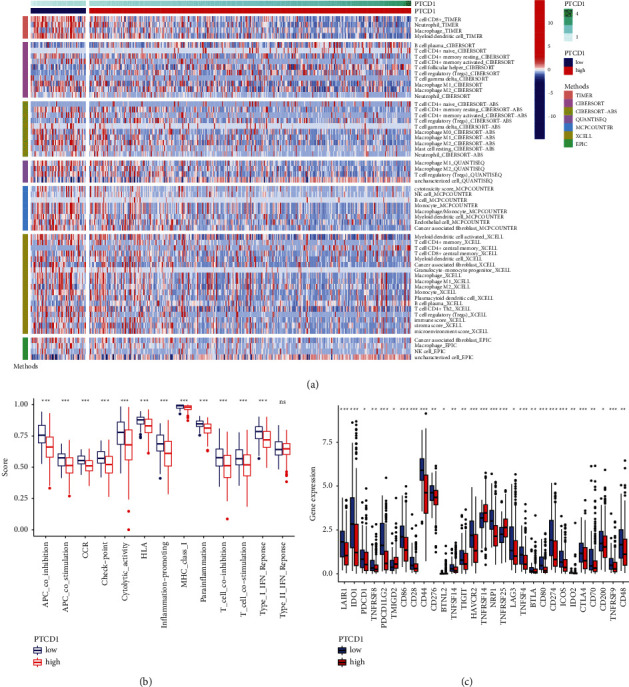
The immune infiltration (a), immune function (b), and immune checkpoint (c) of the high- and low-PTCD1 group for BLCA patients in the TCGA cohorts. ^*∗*^*P* < 0.05; ^*∗∗*^*P* < 0.01; and ^*∗∗∗*^*P* < 0.001.

We summed up the results of 405 BLCA cases computed with various algorithm and compared all the immune cell subtypes in two groups. The proportion of partial infiltrating cell subtypes was obviously different between the two groups, among which mostly myeloid dendritic cell, T cell CD8^+^, neutrophil, macrophage M1, cancer-associated fibroblast, monocyte, myeloid dendritic cell, and macrophage M2 had a higher infiltration scale in the low-PTCD1 group, while T cell regulatory (Tregs), T cell follicular helper, and other types had a lower proportion (Figure 8(a), all *P* < 0.05).

The immune functions of high-PTCD1 group and low-PTCD1 group were analyzed, respectively, using “GSVA” and “GSEABase” package in R software, and it was found that almost immune-related functions (APC coinhibition, APC costimulation, CCR, checkpoint, cytolytic activity, HLA, inflammation promoting, MHC class I, para-inflammation, T cell coinhibition, T cell costimulation, and Type I IFN response) in the low-PTCD1 group were markedly activated (Figure 8(b), all *P* < 0.05), indicating a significant change of immunophenotype in the low-PTCD1 group. We further explored the expressions of immune checkpoint-related biomarkers in two groups and found that some markers (LAIR1, IDO1, PDCD1, TNFRSF8, PDCD1LG2, CD86, CD28, CD44, CD276, TNFSF14, TIGIT, HAVCR2, NRP1, LAG3, TNFSF4, BTLA, CD80, CD274, ICOS, IDO2, CTLA4, CD70, CD200, TNFRSF9, and CD48) in the low-PTCD1 group were upregulated, and some markers (TMIGD2, TNFRSF14, and TNFRSF25) were downregulated, presenting the presence of immunosuppressive and exhausted phenotypes in the low-PTCD1 group (Figure 8(c), all *P* < 0.05). According to the above analysis, we found that two groups had a remarkable different pattern of immune infiltration, which might lead to distinct survival benefits.

### 3.9. The Association between PTCD1 Expression and m6A Regulatory Genes

**Figure 9:**
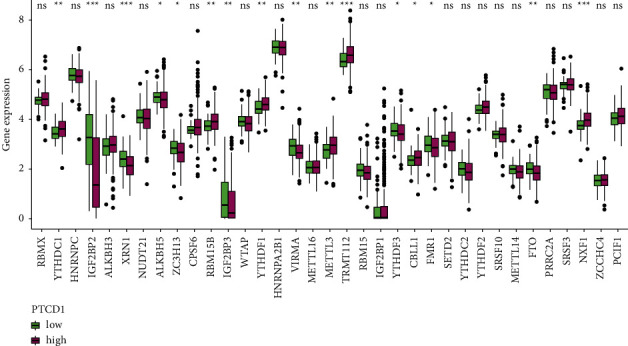
The m6A-related genes of the high- and low-PTCD1 group for BLCA patients in the TCGA cohorts. ^*∗*^*P* < 0.05; ^*∗∗*^*P* < 0.01; and ^*∗∗∗*^*P* < 0.001.

The expressions of m6A-related genes in two groups were analyzed and found that IGF2BP2, XRN1, ALKBH5, ZC3H13, IGF2BP3, VIRMA, YTHDF3, FMR1, and FTO in the low-PTCD1 group were upregulated, and YTHDC1, RBM15 B, YTHDF1, METTL3, TRMT112, CBLL1, and NXF1 were downregulated, indicating some genetic epigenetic changes in the low-PTCD1 group (Figure 9, all *p* less than 0.05). Based on the above results, we found that patients had significantly different m6A patterns in two groups, which may lead to different survival outcomes.

## 4. Discussion

PTCD1 is a mitochondrial matrix protein containing eight PPR domains [17]. Different from other PPR domain proteins, PTCD1 is a low-abundance protein correlated with leucine tRNAs and precursor RNAs that contain leucine tRNAs [17]. Previous studies showed that PTCD1 is straightly linked to and exhausted leucine mitochondrial tRNAs, restraining the translation of mitochondrial-encoded proteins and inhibiting oxidative phosphorylation in the mitochondria [17, 23]. Its abnormal expression may lead to many diseases, such as metabolic dysfunction, obesity, and neoplasms [14, 21, 24–26]. However, the implication of its expression in prognosis and diagnosis of patients with BLCA remains unclear. In this research, we attempted to address the role of PTCD1 in BLCA for the first time.

First, we analyzed the association between the transcriptional level of PTCD1 in BLCA and clinicopathological characteristics through TCGA database. The results revealed that PTCD1 expression in bladder cancer tissues gradually reduced with the growth of stage and grade. HPA database analysis found that the expression of PTCD1 protein in BLCA was lower than that in normal bladder tissue, which in high-grade tumors was lower than that in low-grade tumors. Kaplan–Meier curves indicated that downregulation of PTCD1 led to poor prognosis of BLCA, which was reflected in the late clinical features of cancer pathological staging and lymph node metastasis. Multivariate Cox regression analysis further verified that low-PTCD1 expression may serve as an independent prognostic factor for OS in patients with BLCA. These results suggest that PTCD1 exerts a tumor-suppressive role in BLCA. In this research, we noticed that M stage indicated the distant metastasis, but there was no effect on the expression of PTCD1. Combined with follow-up analyses, PTCD1 may influence cancer progression mainly by regulation of the immune microenvironment, but this might not enhance distant metastasis of tumor cells. When analyzing the basic characteristics of patients, all patients in the low-expression group belong to high-grade tumors, which may indicate that PTCD1 expression is a factor in predicting tumor grade.

The GO analysis indicated that PTCD1-related DEGs was highly abundant in some terms including “extracellular matrix organization,” “neutrophil degranulation,” “cell-matrix adhesion,” and “cell-substrate junction.” Genetic and epigenetic alterations might cause changed molecular pathways concerned tumor procession and, afterwards, force bladder cancer cells towards epithelial-to-mesenchymal transition (EMT) [27, 28]. EMT confers complex reprogramming of sessile, nonmotile urothelial cells with loss of apical polarity and increased migratory capacity [29–31]. Molecular regulators regulate the transition from epithelium to mesenchyme, enabling some urothelial cancer cells to acquire mesenchymal features with the ability to self-renew, evade immune mechanisms, and infiltrate the surrounding basement membrane [29, 32]. Researchers reported that EMT coordinately regulated maintenance of cancer stemness, drug resistance, angiogenesis, and muscle invasion/metastasis, and had been proofed to be a major characteristic [33–35]. Successful elimination of subpopulation of UroCSCs and its differentiated progenies through targeted treatment of EMT and its core partners can clinically reverse EMT, prevent tumor recurrence, and prolong patient survival [36, 37].

Additionally, these genes uncovered by KEGG pathway analysis are remarkably related to PI3K-Akt signaling pathway, focal adhesion, proteoglycans in cancer, ECM-receptor interaction, and oxidative phosphorylation. Regarding bladder cancer, constitutive activation of PI3K/AKT pathway was observed in up to 40% of tumors [38], which pathway combined a serial of external signals to regulate downstream signaling involved in the cell growth, differentiation, angiogenesis, and protein synthesis, ultimately resulting in bladder carcinogenesis, metastatic potential, and therapy resistance [39–43]. The extracellular matrix (ECM) comprises noncellular components playing an important part in the cell behaviors of regulation [44], which dynamically changes in tumor microenvironment and has a crucial role in tumor progression [45]. Tumor cell growth, migration, and apoptosis via regulation of receptor interaction were reported to be related to the extracellular matrix protein 1 (ECM1) expression, which was considered as a predictive parameter in the carcinogenesis and postoperative recurrence of BLCA [46].

One of the factors affecting the occurrence and progression of tumor is tumor mutation burden (TMB) [47]. Some genes involved in TMB have been reported to be of predictive accuracy in OS among BLCA patients [47–49]. In the present work, PTCD1 expression level was highly impacted by the mutation rate of same cancer-related genes, which were of high mutation probability in BLCA. The relationship between mutant genes and PTCD1 expression implies that PTCD1 may play a regulatory part in BLCA, which is needed to be validated by further studies.

The tumor immune cell infiltration is of essential importance for the prognosis of bladder cancer and interferes the response to immunotherapy [50–52]. Several studies have demonstrated the importance of tumor-infiltrating immune cells and other immune molecules (including myeloid dendritic cell, T cell, neutrophil, and macrophage) in the prognosis of bladder cancer [53–56]. Accordingly, infiltration rates of immune cells with the low- and high-PTCD1 expression levels were further analyzed, suggesting that PTCD1 expression was negatively associated with immune cell infiltration, especially for myeloid dendritic cell, T cell, neutrophil, and macrophage, which was consistent with previous work. The level of immune checkpoint molecules was determined to obtain a deeper understanding into the immune landscape of BLCA. Heterogeneous expression of immune checkpoint proteins in the immune microenvironment of BLCA was discovered by the correlation between its expression and PTCD1 expression. Many antigens presentation-related functions were found by the enrichment score analysis of immune-related pathways. For instance, in the low-PTCD1 group, functions including coinhibition and costimulation of APC, CCR, checkpoint, cytolytic activity, HLA, inflammation promoting, MHC class I, para-inflammation, coinhibition, costimulation of T cell, and Type I IFN response were observed. Taken together, we assume that PTCD1 level may indirectly affect the occurrence and development of BLCA via immune cell.

RNA N6-methyladenosine (m6A) modification as the most abundant mRNA modification has attracted much attention recently [57]. The modification of m6A plays a role in the regulation of occurrence and development of tumors by modulating expressions of key genes, becoming the hot spot of research recently [58]. It was reported by previous research that the development of BLCA may be affected by the abnormal modification of m6A methylation [59–62]. The expression levels of 16 regulators related to m6A found in this work greatly depend on the PTCD1 expression level of BLCA. Reasonably, downregulation of PTCD1 in bladder cancer likely causes the abnormal regulation of m6A-related regulators, where more studies need to be done to prove the hypothesis.

In conclusion, PTCD1 was downregulated in BLCA, which was greatly associated with the poor clinical features and prognosis. Further, our data suggested that PTCD1 may participate in the immunological functioning and immune cell infiltration and might play certain roles in tumor progression and metastasis, which could be acted as a potential prognostic target of BLCA. Likewise, there are few limitations existing in this work. First, more long-term survival data of clinical samples are required to support our findings because of the retrospective data and the limitations of clinical information from TCGA. Second, the absence of PTCD1 protein expression level in tumor specimens should be further detected. Thirdly, potential mechanisms underlying the effects of PTCD1 on the clinical outcomes in patients with BLCA were not fully explored. Last but not the least, more studies are needed to investigate the interplay between PTCD1 expression and immune infiltration.

## 5. Conclusions

In this work, the role of PTCD1 in BLCA was explored from the perspectives of clinical values and potential mechanisms of PTCD1. Downregulation of PTCD1 is likely related to BLCA progression. PTCD1 may play some certain parts in cell invasion and metabolic and immune microenvironment of BLCA. All in all, PTCD1 could act as a potential predictor for diagnosis and prognosis and novel therapeutic target in BLCA.

## Figures and Tables

**Table 1:** The relationship between the expression of PTCD1 and various clinicopathological variables in the TCGA database.

Characteristics	PTCD1 expression	PTCD1 expression	Low	High	*P*-value
Total		405	59	346	
Age (years)	≤65	160	25 (6.2%)	135 (33.3%)	0.626
	>65	245	34 (8.4%)	211 (52.1%)	
Sex	Male	298	44 (10.9%)	254 (62.7%)	0.851
	Female	107	15 (3.7%)	92 (22.7%)	
Pathological stage	Stage I	2	0 (0%)	2 (0.5%)	0.001
	Stage II	129	7 (1.7%)	122 (30.1%)	
	Stage III	139	23 (5.7%)	116 (28.6%)	
	Stage IV	133	29 (7.2%)	104 (25.7%)	
Histologic grade	High grade	382	59 (14.6%)	323 (79.8%)	0.057
	Low grade	20	0 (0%)	20 (4.9%)	
T stage	T1	3	0 (0%)	3 (0.7%)	0.193
	T2	118	12 (3.0%)	106 (26.2%)	
	T3	193	35 (8.6%)	158 (39.0%)	
	T4	57	11 (2.7%)	46 (11.4%)	
N stage	N0	235	29 (7.2%)	206 (50.9%)	0.031
	N1	46	12 (3.0%)	34 (8.4%)	
	N2	75	16 (4.0%)	59 (14.6%)	
	N3	7	0 (0%)	7 (1.7%)	
M stage	M0	195	26 (6.4%)	169 (41.7%)	0.648
	M1	11	2 (0.5%)	9 (22.2%)	
Survival status	Alive	249	27 (6.7%)	222 (54.8%)	0.007
	Dead	156	32 (7.9%)	124 (30.2%)	
PTCD1 level (mean ± SD)		1.83 ± 0.75	0.95 ± 0.12	1.98 ± 0.71	<0.0001

SD: standard deviation. *P* < 0.05 are shown in bold.

## Data Availability

Publicly available datasets were analyzed in this study. HPA database and TCGA database belong to public databases. Users can download relevant data for free for research and publish relevant articles.

## Supplementary Materials

Supplementary Materials
*Supplementary File 1*. The differential expression genes between low-PTCD1 group and high-PTCD1 group. *Supplementary File 2*. The results of GO enrichment analysis and KEGG pathway analysis.
